# Effects of dietary methionine on breast muscle growth, myogenic gene expression and IGF-I signaling in fast- and slow-growing broilers

**DOI:** 10.1038/s41598-017-02142-z

**Published:** 2017-05-15

**Authors:** Chao Wen, Xueying Jiang, Liren Ding, Tian Wang, Yanmin Zhou

**Affiliations:** College of Animal Science and Technology, Nanjing Agricultural University, Nanjing, 210095 China

## Abstract

This study investigated the responses of fast- (FG) and slow- (SG) growing broilers to dietary methionine (Met) status. The broilers were subjected to low (LM, 0.38 and 0.28 g/100 g), adequate (AM, 0.51 and 0.42 g/100 g) and high (HM, 0.65 and 0.52 g/100 g) Met during 1–21 and 22–42 d, respectively. Compared with the LM diets, the AM and HM diets increased body weight gain only in the FG broilers. The HM diets increased breast muscle yield only in the FG broilers, although insulin-like growth factor-I (IGF-I) concentration was increased in both strains of broilers. The HM diets increased mRNA levels of myogenic regulatory factors (MRF4, Myf5) and myocyte enhancer factor 2 (MEF2A and MEF2B) in the FG broilers, and increased MEF2A and decreased myostatin mRNA level in the SG broilers. Extracellular signal-regulated kinase (ERK) phosphorylation of breast muscle was increased by the HM diets in both strains of broilers, but mechanistic target of rapamycin (mTOR) phosphorylation was increased by the AM and HM diets only in the FG broilers. These results reflect a strain difference in broiler growth and underlying mechanism in response to dietary Met.

## Introduction

Methionine (Met) is an essential amino acid for protein synthesis and a precursor of S-adenosylmethionine, the methyl donor for DNA methylation^1^. As the first limiting amino acid in broiler diets, Met has been demonstrated to affect growth performance and breast meat yield in broilers^2^. Therefore, it is important to provide adequate Met to optimise broiler growth and production. Growth performance and breast muscle yield are often used to estimate Met requirement of broilers, which has been reported to depend on gender, age, nutrient density, rearing environment, etc^3, 4^. However, few studies have compared Met requirement between broiler strains differing in growth rate. The Met contents given in the NRC (1994)^5^ are generally minimum levels that satisfy general productive activities of broilers without strain being considered. In general, fast-growing (FG) broilers have superior growth performance and breast muscle yield to slow-growing (SG) broilers^6, 7^. Thus, it can be implied that the responses of growth performance and breast muscle yield to dietary Met status may be different between FG and SG broilers, which needs further elucidation.

Skeletal muscle development is regulated by myogenic regulatory factors (including MyoD, Myf5, MyoG, and MRF4), myocyte enhancer factor 2 (including MEF2A, B, C, and D), myostatin (MSTN) and insulin-like growth factor-I (IGF-I) signaling pathways^8, 9^. The downstream targets of IGF-I include extracellular signal-regulated kinase (ERK), mechanistic target of rapamycin (mTOR) and forkhead box O (FoxO), which play an important role in cell proliferation and protein synthesis^10, 11^. Our laboratory recently reported that MSTN, mTOR and FoxO4 were involved in the enhancement of breast muscle growth by Met in broilers with lower but not high hatching weight^12^, suggesting that the responses to dietary Met were dependent on hatching weight of chicks. However, whether these responses are affected by broiler strain remains unclear. The FG and SG broilers may have different Met requirements^13^, so they may have different responses of breast muscle yield and associated gene and protein expression to dietary Met status. Therefore, the objective of this study was to compare the effects of dietary Met on growth performance, breast muscle yield and expression of myogenic genes and proteins associated with IGF-I signaling pathway in FG and SG chickens.

## Results

### Growth performance

Higher body weight gain and feed intake as well as lower feed conversion ratio were observed (*P* < 0.001) in the FG broilers than in the SG broilers (Table 1). Compared with the LM diets, the AM and HM diets increased (*P* < 0.05) body weight gain and the AM diets increased (*P* < 0.05) FI in the FG broilers, but no difference was found in the SG broilers. There was no Met effect on feed conversion ratio within each strain.

**Table 1:** Effects of dietary methionine on growth performance of broilers from 1 to 42 d of age. Results are presented as means and pooled SEM (n = 6).

	Body weight gain (kg)	Feed intake (kg)	Feed conversion ratio
Strain			
FG	2.01	3.94	1.96
SG	1.14	2.68	2.36
Met			
LM	1.49^b^	3.19^b^	2.20
AM	1.62^a^	3.40^a^	2.14
HM	1.62^a^	3.34^a^	2.13
Strain×Met			
FG+LM	1.91^b^	3.80^b^	1.99^b^
FG+AM	2.07^a^	4.08^a^	1.98^b^
FG+HM	2.06^a^	3.94^ab^	1.91^b^
SG+LM	1.07^c^	2.71^c^	2.41^a^
SG+AM	1.17^c^	2.58^c^	2.31^a^
SG+HM	1.17^c^	2.74^c^	2.34^a^
SEM	0.02	0.03	0.02
*P* value			
Strain	<0.001	<0.001	<0.001
Met	0.004	0.010	0.173
Strain×Met	0.777	0.358	0.453

Mean values within a column with unlike superscript letters were significantly different (*P* < 0.05).

### Breast muscle weight and insulin-like growth factor-I concentration

The FG broilers had higher (*P* < 0.001) absolute and relative breast muscle weight than the SG broilers, but the IGF-I concentration did not differ between the two strains of broilers (Table 2). Compared with the LM diets, the HM diets increased (*P* < 0.05) absolute and relative breast muscle weight only in the FG broilers, but the AM and HM diets increased (*P* < 0.05) IGF-I concentration of breast muscle in both strains of broilers.

**Table 2:** Effects of dietary methionine on breast muscle weight and insulin-like growth factor-I (IGF-I) concentration of broilers at 42 d of age.

	Breast muscle weight		IGF-I (ng/mg protein)
	Absolute (g)	Relative (g/kg BW)	
Strain			
FG	396	181.7	13.63
SG	132	97.5	14.10
Met			
LM	228^b^	130.3^b^	12.72^c^
AM	265^ab^	140.7^ab^	14.08^b^
HM	300^a^	147.8^a^	14.80^a^
Strain×Met			
FG+LM	341^b^	167.9^b^	12.33^d^
FG+AM	393^ab^	180.4^ab^	13.71^bc^
FG+HM	456^a^	196.9^a^	14.85^a^
SG+LM	116^c^	92.7^c^	13.10^cd^
SG+AM	137^c^	101.0^c^	14.45^ab^
SG+HM	144^c^	98.7^c^	14.75^ab^
SEM	9	2.6	0.14
*P* value			
Strain	<0.001	<0.001	0.103
Met	0.020	0.039	<0.001
Strain×Met	0.191	0.180	0.310

Results are presented as means and pooled SEM (n = 6). Mean values within a column with unlike superscript letters were significantly different (*P* < 0.05).

### Gene expression

The main effects showed that the FG broilers had higher (*P* < 0.05) mRNA expression of MRF4, Myf5, MEF2B and MEF2D in breast muscle than the SG broilers (Table 3). Compared with the LM diets, the HM diets increased (*P* < 0.05) mRNA levels of MRF4, Myf5, MEF2A and MEF2B in the FG broilers, and increased MEF2A and decreased MSTN mRNA level in the SG broilers. There were no differences in mRNA levels of MyoD, MyoG or MEF2C among treatments.

**Table 3:** Effects of dietary methionine on relative mRNA levels in breast muscle of broilers at 42 d of age.

	MSTN	MyoD	MyoG	MRF4	Myf5	MEF2A	MEF2B	MEF2C	MEF2D
Strain									
FG	1.03	0.98	0.92	1.01	0.96	0.93	1.03	0.92	1.03
SG	1.21	0.72	1.04	0.65	0.64	0.83	0.64	0.69	0.54
Met									
LM	1.56^a^	0.71	0.80	0.59^b^	0.53^b^	0.64^b^	0.65^b^	0.62	0.56
AM	1.02^b^	0.85	1.01	0.81^ab^	0.78^b^	0.94^a^	0.75^b^	0.89	0.76
HM	0.78^b^	0.98	1.13	1.09^a^	1.10^a^	1.06^a^	1.11^a^	0.93	1.04
Strain×Met									
FG+LM	1.42^ab^	0.74	0.70	0.60^b^	0.50^b^	0.69^bc^	0.78^bc^	0.73	0.70^ab^
FG+AM	1.00^ab^	1.00	1.00	1.00^ab^	1.00^ab^	1.00^ab^	1.00^ab^	1.00	1.00^ab^
FG+HM	0.68^b^	1.19	1.06	1.43^a^	1.39^a^	1.08^a^	1.31^a^	1.04	1.40^a^
SG+LM	1.71^a^	0.69	0.91	0.57^b^	0.55^b^	0.58^c^	0.51^c^	0.50	0.42^b^
SG+AM	1.05^ab^	0.70	1.03	0.63^b^	0.55^b^	0.87^abc^	0.50^c^	0.77	0.52^b^
SG+HM	0.88^b^	0.78	1.20	0.75^b^	0.81^b^	1.04^a^	0.90^bc^	0.81	0.69^ab^
SEM	0.10	0.09	0.06	0.07	0.06	0.04	0.05	0.07	0.10
*P* value									
Strain	0.365	0.186	0.325	0.014	0.020	0.941	0.001	0.105	0.035
Met	0.016	0.547	0.111	0.015	0.008	0.001	0.003	0.148	0.203
Strain×Met	0.854	0.741	0.809	0.137	0.153	0.236	0.689	0.998	0.727

Results are presented as means and pooled SEM (n = 6). Mean values within a column with unlike superscript letters were significantly different (*P* < 0.05).

### Protein expression

**Figure 1:**
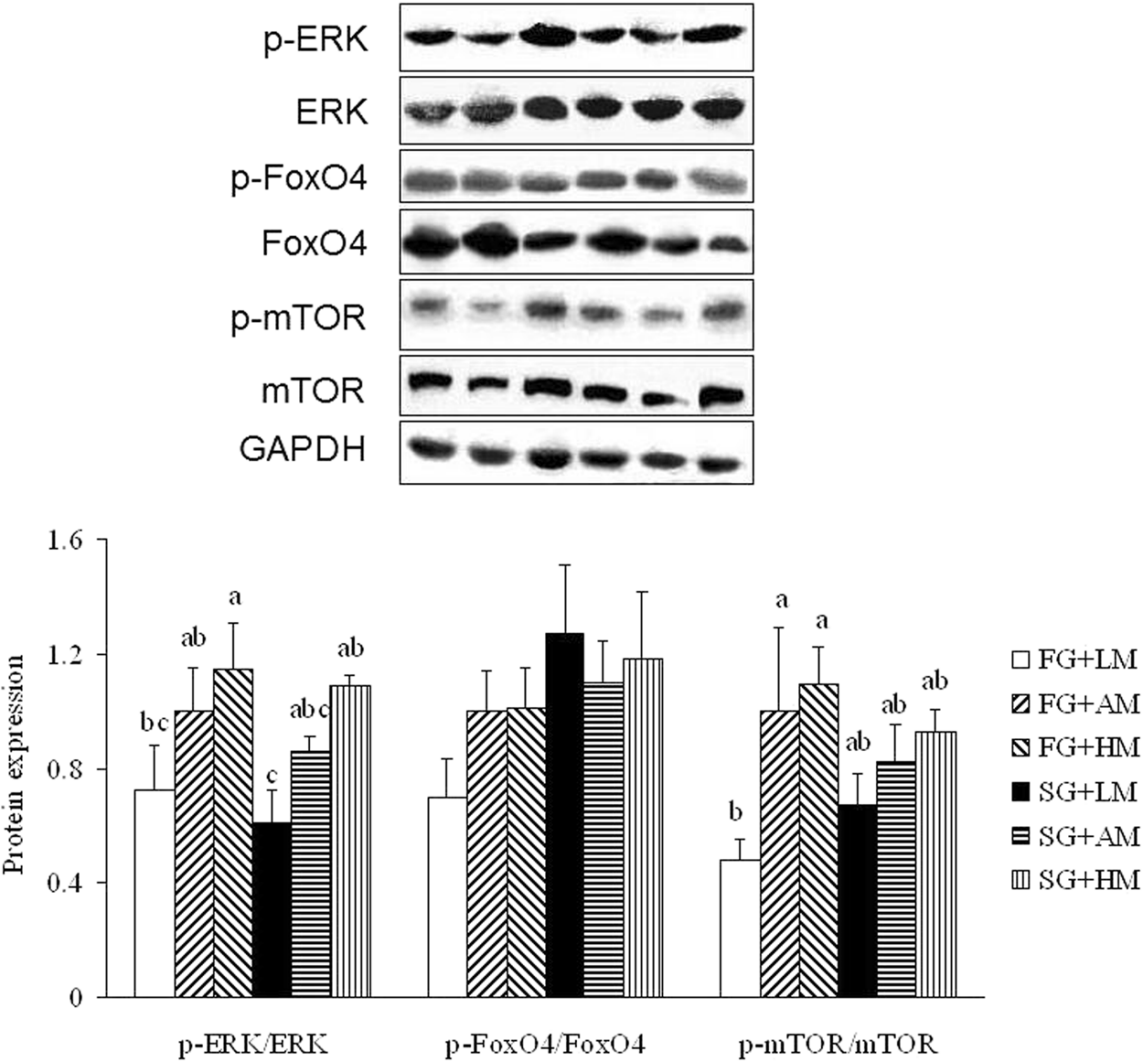
Effects of dietary methionine on protein expression in breast muscle of broilers at 42 d of age. Results are presented as means ± SEM (n = 6). Bars marked with different letters are significantly different at *P* < 0.05. Full-length blots are shown in Supplementary Figure S1.

There was no strain difference in phosphorylation of ERK, mTOR or FoxO4 in breast muscle, although FoxO4 phosphorylation tended (*P* = 0.066) to be higher in the FG broilers (Fig. 1, full-length blots are shown in Supplementary Figure S1). Compared with the LM diets, the HM diets increased (*P* < 0.05) ERK phosphorylation of breast muscle in both strains of broilers, and the AM and HM diets increased (*P* < 0.05) mTOR phosphorylation of breast muscle in the FG broilers. The effect of Met on FoxO4 phosphorylation of breast muscle was not significant within each strain.

## Discussion

As expected, the FG broilers had superior growth performance and breast muscle yield to the SG broilers. However, there was no significant difference in the IGF-I concentration of breast muscle between the strains, supporting the previous study that reported similar IGF-I mRNA expression in breast muscle between FG and SG chickens when they were fed the same diet, although significant differences were observed in live weight and breast muscle weight^14^. It was reported that body weight and circulating IGF-I levels were higher in FG chickens than in SG chickens from 1 to 6 weeks^15^. Thus, the present results implied that the strain differences in growth performance and breast muscle weight might be associated with circulating but not autocrine/paracrine IGF-I levels.

The FG broilers had higher mRNA expression of MRF4, Myf5, MEF2B and MEF2D in breast muscle than the SG broilers, indicating that these genes might be involved in the faster breast muscle development for the FG broilers. It has been demonstrated that MyoD and Myf5 are required for myogenic determination whereas MyoG and MRF4 are important for terminal differentiation^16^, and MEF2 family also play an important role in regulating muscle differentiation^17^. Thus, our findings suggest that the FG broilers may enhance myogenic determination and differentiation by upregulating the expression of MRF4, Myf5, MEF2B and MEF2D. Yin *et al*.^18^ also found greater mRNA abundance of Myf5 and MRF4 in lines of chickens selected for high body weight than in those selected for low body weight at 28 d of age.

There was no strain difference in phosphorylation of ERK, FoxO4 or mTOR, although FoxO4 phosphorylation tended to be higher in the FG broilers, suggesting that the faster breast muscle development of the FG broilers might be regulated by some other signaling pathways, which deserves further investigation. Similarly, Wang *et al*.^7^ found no difference in mTOR mRNA expression in the pectoralis major muscle between the FG and SG broilers at 63 or 105 d of age.

This study demonstrated that Met supplementation improved growth performance and breast muscle weight of the FG broilers as previously reported^19^, which might be due to simultaneously increased IGF-I concentration. Dietary Met has been reported to affect IGF-I concentration in breast muscle of broilers^20^. However, the growth performance and breast muscle weight did not differ between the broilers fed the AM and HM diets, which did not agree with the data of Ahmed and Abbas^21^, who reported that dietary Met higher than NRC (1994)^5^ increased body weight gain and breast muscle weight of broilers compared with the control. The discrepancy may be due to higher finisher Met level (0.44%) in the AM diets in our study than that (0.35%) in the control diet in Ahmed and Abbas^21^. Dietary treatments did not affect growth performance or breast muscle weight in the SG broilers, although increased IGF-I concentration was observed. This implies that the SG broilers may be less sensitive to Met supplementation probably due to lower Met requirement for growth and muscle protein accretion^22^.

In this study, the HM diets resulted in strain-dependent changes in myogenic gene expression in breast muscle compared with the LM diets, with MRF4, Myf5, MEF2A and MEF2B upregulated in the FG broilers but MEF2A upregulated and MSTN downregulated in the SG broilers. This implied that dietary Met affected myogenic determination and differentiation in a strain-dependent way, particularly in the FG broilers. We previously found that increasing dietary Met levels increased Myf5 and MEF2B mRNA expression and decreased MSTN mRNA expression in broilers^23^. It was reported that methylation status of MSTN gene was negatively correlated with the gene expression in skeletal muscle of broilers fed various levels of Met^24^. The effect of dietary Met on methylation of DNA has been reported^25^. Thus it can be inferred that the changes in myogenic gene expression in the FG and SG broilers may be attributed to altered DNA methylation.

The ERK1/2 signaling pathway is required for myoblast terminal differentiation^26^, and mTOR and FoxO pathways play an important role in muscle protein synthesis and degradation, respectively^27^. In this study, the HM diets increased ERK phosphorylation of breast muscle in both strains of broilers compared with the LM diets, but increased mTOR phosphorylation only in the FG broilers, implying that Met may affect myoblast differentiation and protein synthesis in breast muscle in a strain-related manner. The changes of myogenic gene expression induced by the HM diets described above may be attributed to increased ERK phosphorylation as shown by Li *et al*.^28^, who found that the expression of muscle regulatory factors and ERK activation were suppressed during the inhibition of myogenic differentiation by hypoxia, and increasing ERK activity by forced expression of mitogen-activated protein kinase kinase 1 could partly reverse the inhibition of myogenic differentiation by hypoxia. Increased mTOR phosphorylation may also play a role in regulating myogenic gene expression as reviewed recently^29^. We previously reported that Met supplementation increased mTOR phosphorylation in breast muscle of broilers with lower hatching weight^12^. No difference in FoxO4 phosphorylation within each strain suggested that muscle proteolysis was not affected by dietary Met.

In conclusion, enhanced myogenic gene expression may be involved in the superior growth performance and breast muscle yield of the FG broilers. The HM diets improved growth performance and breast muscle yield of the FG broilers probably by regulating myogenic gene expression, IGF-I synthesis and phosphorylation of ERK and mTOR, but the SG broilers were less sensitive to Met supplementation.

## Methods

All animal handling procedures were performed in strict accordance with guide for the Care and Use of Laboratory Animals central of the Nanjing Agricultural University, and the protocol was approved by the Institutional Animal Care and Use Committee of the Nanjing Agricultural University.

### Bird husbandry, diets, and experimental design

The Arbor Acres broiler was selected as the FG strain, and Partridge Shank chicken, a typical indigenous meat-type strain in China, was selected as the SG strain. A total of 180 broilers from each strain were obtained from a local hatchery and raised from 1 to 42 d of age. The broilers were divided into a 2 × 3 factorial arrangement of treatments with six replicate cages of ten broilers per treatment. Diets were formulated according to the NRC (1994)^5^ to contain low (LM, 0.38 and 0.28 g/100 g), adequate (AM, 0.51 and 0.42 g/100 g) and high (HM, 0.65 and 0.52 g/100 g) Met during 1–21 and 22–42 d, respectively. The LM diets contained no supplemental DL-Met, and the AM and HM diets were formulated by supplementing DL-Met (99%; Adisseo Inc.) in the LM diets at the expense of maize gluten meal (Table 4). Dietary crude protein content was determined according to the procedures of AOAC (2000)^30^, and amino acid composition was determined using an L-8900 Amino Acid Analyzer (Hitachi) as previously described^31^. Methionine and cystine were analyzed as Met sulfoxide and cysteic acid after performic acid oxidation. Chicks were allowed free access to mash feed and water in 3-layer cages in a temperature-controlled room with a 23 L:1D lighting program. The temperature of the room was maintained at 32 to 34 °C for the first 3 d and then reduced by 2 to 3 °C per week to a final temperature of 20 °C. Feed intake was recorded by replicate cage at 21 and 42 d of age. At 42 d of age, birds were weighed after feed deprivation for 12 h to calculate body weight gain and feed conversion ratio (feed intake:weight gain).

**Table 4:** Ingredients and nutrient composition of experimental diets (as-fed basis).

Item	1–21 d			22–42 d		
	LM	AM	HM	LM	AM	HM
Ingredient (g/100 g)						
Maize	57.09	57.09	57.09	61.08	61.08	61.08
Soybean meal	31.37	31.37	31.37	28.7	28.7	28.7
Maize gluten meal	3.71	3.56	3.41	1.70	1.57	1.44
Soybean oil	3.00	3.00	3.00	4.01	4.01	4.01
Dicalcium phosphate	2.00	2.00	2.00	1.60	1.60	1.60
Limestone	1.20	1.20	1.20	1.30	1.30	1.30
L-Lysine, HCl	0.33	0.33	0.33	0.31	0.31	0.31
DL-Methionine	0	0.15	0.30	0	0.13	0.26
NaCl	0.30	0.30	0.30	0.30	0.30	0.30
Vitamin and mineral mix^1^	1.00	1.00	1.00	1.00	1.00	1.00
Analysed composition (g/100 g)^2^						
Crude protein	21.16	21.32	21.27	19.04	19.07	19.12
Lysine	1.14	1.18	1.14	1.03	1.06	1.08
Methionine	0.38	0.51	0.65	0.28	0.42	0.52
Total sulphur amino acids	0.73	0.89	1.00	0.61	0.75	0.88

^1^The premix provided the following nutrients per kilogram of diet: retinyl acetate, 10000 IU; cholecalciferol, 3000 ICU; all-rac-α-tocopherol acetate, 30 IU; menadione, 1.3 mg; thiamin, 2.2 mg; riboflavin, 8 mg; nicotinamide, 40 mg; choline chloride, 600 mg; calcium pantothenate, 10 mg; pyridoxine·HCl, 4 mg; biotin, 0.04 mg; folic acid, 1 mg; cobalamin, 0.013 mg; Fe (as FeSO_4_.H_2_O), 80 mg; Cu (as CuSO_4_.5H_2_O), 8 mg; Mn (as MnSO_4_.H_2_O), 110 mg; Zn (as ZnO), 65 mg; I (as KIO_3_), 1.1 mg; Se (as Na_2_SeO_3_), 0.3 mg. ^2^The nutrient composition was analyzed in triplicate for each diet, and average values were reported.

### Sample collection

At 42 d of age, one male broiler per replicate was selected and weighed after feed deprivation for 12 h. Birds were killed by cervical dislocation. After dissection, the whole breast muscle (pectoralis major and pectoralis minor without bones) was weighed. Then samples were immediately collected from pectoralis major muscles and stored in liquid nitrogen until analysis.

### Measurement of insulin-like growth factor-I in breast muscle

After thawing at room temperature, the breast muscle samples were homogenised with ice-cold physiological saline solution, and then centrifuged at 5000 × g for 10 min at 4 °C to collect the supernatant. Total protein of the supernatant was determined by the Bradford method^32^. The concentration of IGF-I was measured by a commercial chicken-specific ELISA kit (Nanjing Jiancheng Bioengineering Institute) and expressed as ng/mg protein.

### mRNA quantification

The mRNA expression was determined as previously described^33^. Briefly, total RNA of samples was isolated using RNAiso reagent (TaKaRa Biotechnology) and diluted in diethyl pyrocarbonate treated water to appropriate concentration. Then the diluted RNA was immediately reverse transcribed into cDNA with PrimeScript RT reagent Kit (TaKaRa), and the cDNA was quantified using SYBR Premix Ex Taq II (TaKaRa) on ABI 7300 Real-Time PCR System (Applied Biosystems). Optimised cycling conditions were 95 °C for 30 s followed by 40 cycles of 95 °C for 5 s, 60 °C for 31 s, and final dissociation stage of 95 °C for 15 s, 60 °C for 1 min, 95 °C for 15 s and 60 °C for 15 s. The geometric means of glyceraldehyde 3-phosphate dehydrogenase (GAPDH) and β-actin were used to normalise the genes of interest as recommended^34^. The primers for GAPDH, β-actin, MSTN, MyoD, MyoG, and MRF4, Myf5, MEF2A, MEF2B, MEF2C and MEF2D were synthesised as previously reported^23^. Relative mRNA levels (arbitrary units) were calculated on the basis of PCR efficiency and threshold cycle (Ct) values^35^. The mRNA level of each target gene in the FG broilers fed the AM diets was assigned a value of one.

### Western blot

The samples were homogenised in ice-cold RIPA lysis buffer (Beyotime Institute of Biotechnology) and then centrifuged at 12000 × g for 10 min at 4 °C to collect the supernatant. Protein concentration in the supernatant was determined using a bicinchoninic acid protein assay kit (Bioworld Technology, Inc.). The proteins were separated by SDS-PAGE and transferred to polyvinylidene fluoride membrane. Then the membrane was blocked and incubated with appropriate antibodies: mTOR, FoxO4, p-mTOR (Ser2448), p-FoxO4 (Ser193) and p-ERK (Thr202/Tyr204) were purchased from Cell Signaling Technology, Inc., and ERK from Bioworld Technology, Inc. The GAPDH antibody (Bioworld Technology, Inc.) was used as a loading control. After washing, membranes were incubated with a secondary antibody (Rockland Immunochemicals). The bands were visualised by infrared fluorescence using the Odyssey Imaging System (LI-COR) and quantified by Odyssey infrared imaging system software.

### Statistical analysis

Two-way ANOVA was performed to determine the main effects of strain and Met and their interaction using the general linear model procedure of SPSS software (version 16.0; SPSS Inc.). Differences among treatments were examined by Duncan’s multiple range test, which were considered significant at *P* < 0.05. Data are presented as means and standard error of means (SEM).

## Electronic supplementary material

Supplementary Figure S1
