# Supplementary material for: Effects of dietary methionine on breast muscle growth, myogenic gene expression and IGF-I signaling in fast- and slow-growing broilers
Source: Sci Rep. 2017 May 15;7:1924. doi: 10.1038/s41598-017-02142-z (PMC5432508; doi:10.1038/s41598-017-02142-z)

Title: Effects of dietary methionine on breast muscle growth, myogenic gene expression and IGF-I signaling in fast- and slow-growing broilers

Author: Chao Wen, Xueying Jiang, Liren Ding, Tian Wang, Yanmin Zhou

Supplementary Figure S1. Full-length blots for the data in Figure 1.

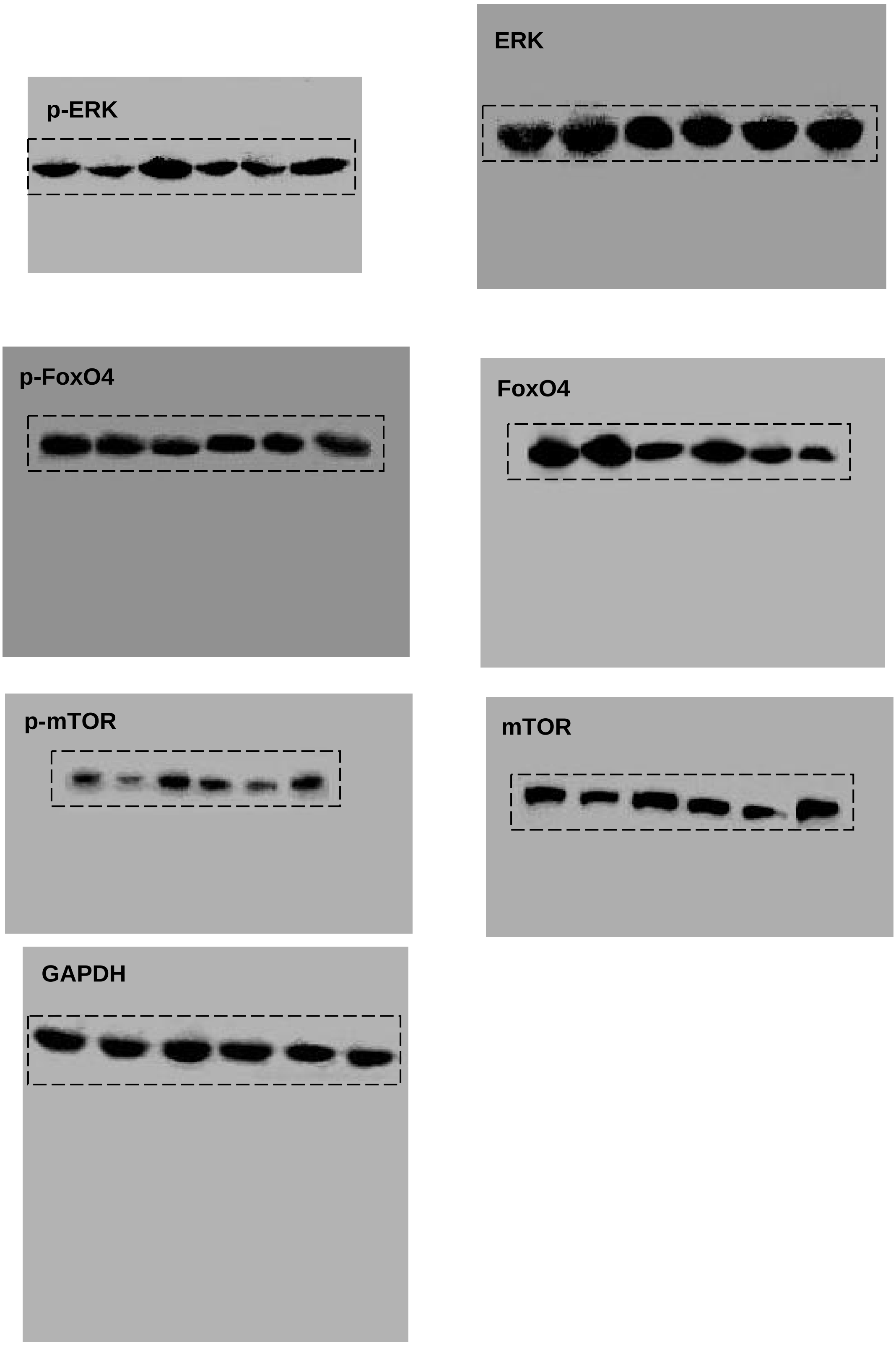

Supplement: Supplementary file 1 — Supplementary Figure S1 [file 41598_2017_2142_MOESM1_ESM.pdf]
